# Targeted metatranscriptomic detection of viruses from floors for simultaneous evaluation of respiratory disease burden and viral variant identification

**DOI:** 10.1128/msphere.00086-26

**Published:** 2026-04-20

**Authors:** Amanda C. Carroll, Aaron Hinz, Alexandra M. A. Hicks, Engluy Khov, Tamara Van Bakel, Evgueni Doukhanine, Michael Fralick, Caroline Nott, Rees Kassen, Nisha Thampi, Laura A. Hug, Derek MacFadden, Alex Wong

**Affiliations:** 1 The Ottawa Hospital Research Institute 10055, Ottawa, Ontario, Canada; 2 McGill University 5620, Montreal, Quebec, Canada; 3 Carleton University 6339, Ottawa, Ontario, Canada; 4 The University of Ottawa 6363, Ottawa, Ontario, Canada; 5 Children’s Hospital of Eastern Ontario, Ottawa, Ontario, Canada; 6 Sinai Health System 518775, Toronto, Ontario, Canada; 7 DNA Genotek 550799 , Ottawa, Ontario, Canada; 8 The University of Toronto, Toronto, Ontario, Canada; 9 The Ottawa Hospital 630825 , Ottawa, Ontario, Canada; 10 University of Waterloo 8430, Waterloo, Ontario, Canada; Tokai Daigaku Igakubu Daigakuin Igaku Kenkyuka, Isehara, Kanagawa, Japan

**Keywords:** environmental surveillance, targeted metatranscriptomics, viruses, hospital

## Abstract

Built environment surveillance is a proven approach for tracking disease burden of some viruses within hospitals and long-term care facilities. However, studies in clinical settings are lacking for simultaneously surveying targets in a built environment using targeted metatranscriptomics. We swabbed six discrete floor locations within an acute care center’s emergency department (ED) in Ottawa, Canada, and sequenced cDNA using a 132 viral taxa panel, identifying viral burden across sampling locations and time. The determined SARS-CoV-2 variant profile across time was matched to provincial variant prevalence. The correlation between metatranscriptomic read abundances and reported cases of influenza A, SARS-CoV-2, and RSV was assessed. We quantified these via qPCR and assessed the correlation of Cq versus metatranscriptomic reads for these viruses. We sequenced a median of 1,302,882 reads per sample from 38 floor swabs collected during peak respiratory viral season (November 2022–February 2023). Diversity of viral communities varied significantly across locations in the ED. SARS-CoV-2 variant abundance shifts matched the changing infection landscape concurrently reported in Ontario. Relationships between targeted metatranscriptomic read ratios and clinical burden were not statistically significant, although we found modest correspondence between qPCR signal and read depth for RSV and SARS-CoV-2. This approach characterized the viral communities and the within-species diversity within an ED. Correlating sequencing-derived data with disease burden for three key respiratory viruses was inconsistent, with the exception of significant correlation between metatranscriptomic reads and Cq data for SARS-CoV-2. We were able to recover the distribution of clinically reported SARS-CoV-2 variants from the floor swab data.

**IMPORTANCE:**

Environmental surveillance is useful for estimating the disease burden for certain viruses. qPCR is commonly used for surveillance of wastewater and built environments, including during the COVID-19 pandemic, but single, multiplexed reaction targets are limited. Targeted metagenomic or metatranscriptomic approaches can accurately quantify microbial populations of interest in an environment, reduce off-target sequencing, and evaluate a broader number of targets than qPCR assays. Here, we assessed the capacity of a targeted viral metatranscriptomic panel to correlate viral abundance in the hospital built environment with key pathogens of interest, including influenza A, RSV, and SARS-CoV-2. Our results suggest that targeted metatranscriptomics may identify viral communities in healthcare facilities, including strain-level detection capability. However, this approach must be validated for its effectiveness in viral surveillance that accurately reflects disease burden. This work contributes to a growing toolkit for pathogen surveillance, a critical endeavor to safeguard against outbreaks of known and emerging pathogens.

## INTRODUCTION

Environmental surveillance has emerged as a powerful complement to patient-based testing for infectious disease monitoring. Pathogen detection from environmental samples such as air, built surfaces, or wastewater can be used as a proxy for disease burden in a human population (1–5). During the COVID-19 pandemic, wastewater provided valuable population-level estimates of disease burden (6). Many jurisdictions used wastewater to guide public health recommendations concerning the duration of stay-at-home orders, the speed of reopening, and the deployment of testing, vaccination sites, or specific restrictions for localized communities experiencing spikes in wastewater signals for the virus (7).

Built environment surveillance has significant potential for predicting and managing infectious disease in congregate settings. Built surfaces, and particularly the floor (8), serve as a “sink” for viruses and bacteria released from human hosts. Aerosols, droplets, skin, and other materials shed from humans eventually end up on the floor, where they remain until the surface is disinfected or the organic materials degrade. As such, detection and quantification of pathogens on built surfaces can provide a useful proxy for clinical disease burden. Viral genetic material can begin to degrade within days of shedding the virus. However, quality sequencing data can still be obtained from samples collected from built surfaces even after suspected degradation has occurred (9, 10). Moreover, given that hospital floors are cleaned regularly (e.g., daily), little viral degradation is expected to occur between cleanings. Multiple studies have shown that influenza A, respiratory syncytial virus (RSV), and severe acute respiratory syndrome coronavirus 2 (SARS-CoV-2) can be detected on built surfaces in hospitals (9, 10), schools, daycares, and homes (11–13). Our previous work has shown that SARS-CoV-2 signal from floors mirrors disease burden in hospitals, long-term care homes (LTCHs), and a university (1–4, 14), and that increases in floor signal often precede clinical outbreaks by several days (2).

Over the past year, there has been substantial interest in expanding environmental sampling beyond SARS-CoV-2 to monitor and predict levels of other key pathogens, such as West Nile virus and monkeypox virus (15). The ideal tool for multi-target environmental surveillance would detect many pathogens and genes of interest simultaneously. At present, environmental surveillance typically uses qPCR to detect one or a few pathogens of interest (16–18). Metagenomic and metatranscriptomic approaches have the potential to provide surveillance for hundreds or thousands of pathogens simultaneously (19, 20). In metagenomic/metatranscriptomic (“meta-‘omic” hereafter) sequencing, all DNA/RNA in a sample is sequenced, in principle allowing for the detection of all pathogens, given sufficient read depth. Metagenomic sequencing has previously been used to characterize microbial communities in the built environment of hospitals (21–23). These studies have shown that pathogens can readily be detected on a variety of surfaces, including floors and bedrails, and that resident microbial lineages can be recovered from surfaces. Further, such sequencing can be used to assess the presence of viral variants and identify the proportions at which they are present within samples (24–26).

An important drawback of meta-‘omics is that target organism sequences may be difficult to detect due to the presence of the genetic material from other organisms: because all DNA/RNA in a sample is sequenced, the majority of sequencing reads may not belong to pathogens of interest, but rather to commensal microorganisms and other organisms in the environment (such as humans). Targeted meta-‘omic sequencing can overcome this limitation. In targeted approaches, tens to thousands of genetic targets are enriched via PCR or hybrid-capture and then sequenced, minimizing the off-target sequencing characteristic of shotgun meta-‘omics (27). Targeted approaches have been shown to increase sensitivity in both clinical (27, 28) and environmental (29, 30) settings.

While the ability of targeted meta-‘omics to detect pathogens in the environment has been established (19, 31, 32), further work is needed before these methods can be appropriately used in surveillance. Targeted meta-‘omics has been used for pathogen detection in wastewater (27), but applicability to more clinically relevant samples has not been demonstrated. Moreover, the degree to which meta-‘omic signal correlates with disease burden has not been established, and there may be potential limitations to these. That is, pathogen sequences must be known in order to be added to the panel; novel taxons or sequences may not be included in these commercial panels in a timely manner, and the enrichment process may be impaired by the accumulation of mutations in the target sequences, creating potential mismatches between target and probe (33). Validating targeted sequencing techniques for built environment samples is critical to determine meaningful relationships between built environment surveillance and population health.

Here, we describe the proof-of-principle application of targeted metatranscriptomics for surveillance of viruses in a healthcare setting.

## RESULTS

We applied the Qiagen xHyb Adventitious Agent Panel to RNA extracted from 38 floor swabs collected over 3 months from six sampling locations within a large tertiary care emergency department (ED). Briefly, these locations included the triage area (TRI) of the hospital emergency department, two waiting rooms (waiting rooms A [WRA] and B [WRB]), the foyer outside WRB (OWB), and the hallways outside of two exam rooms (hallways A [HWA] and B [HWB]). Following bait-capture and sequencing of these samples, we obtained a median of 4,953,607 total reads (range: 2,299,242–30,550,722; Table S1) and 1,302,882 viral reads per sample (range: 331,180–24,509,828; Table S1). The negative control was found to have the lowest number of reads, both total and viral target reads (782,964 and 55,148 reads, respectively); all samples were maintained for analyses. The environmental matrix-free contrived positive control, composed of known amounts (200 copies/µL) of SARS-CoV-2, influenza A, influenza B, and RSV, had the highest number of reads, with 34,814,560 total reads and 30,562,516 viral target reads.

### Viral diversity

**Fig 1:**
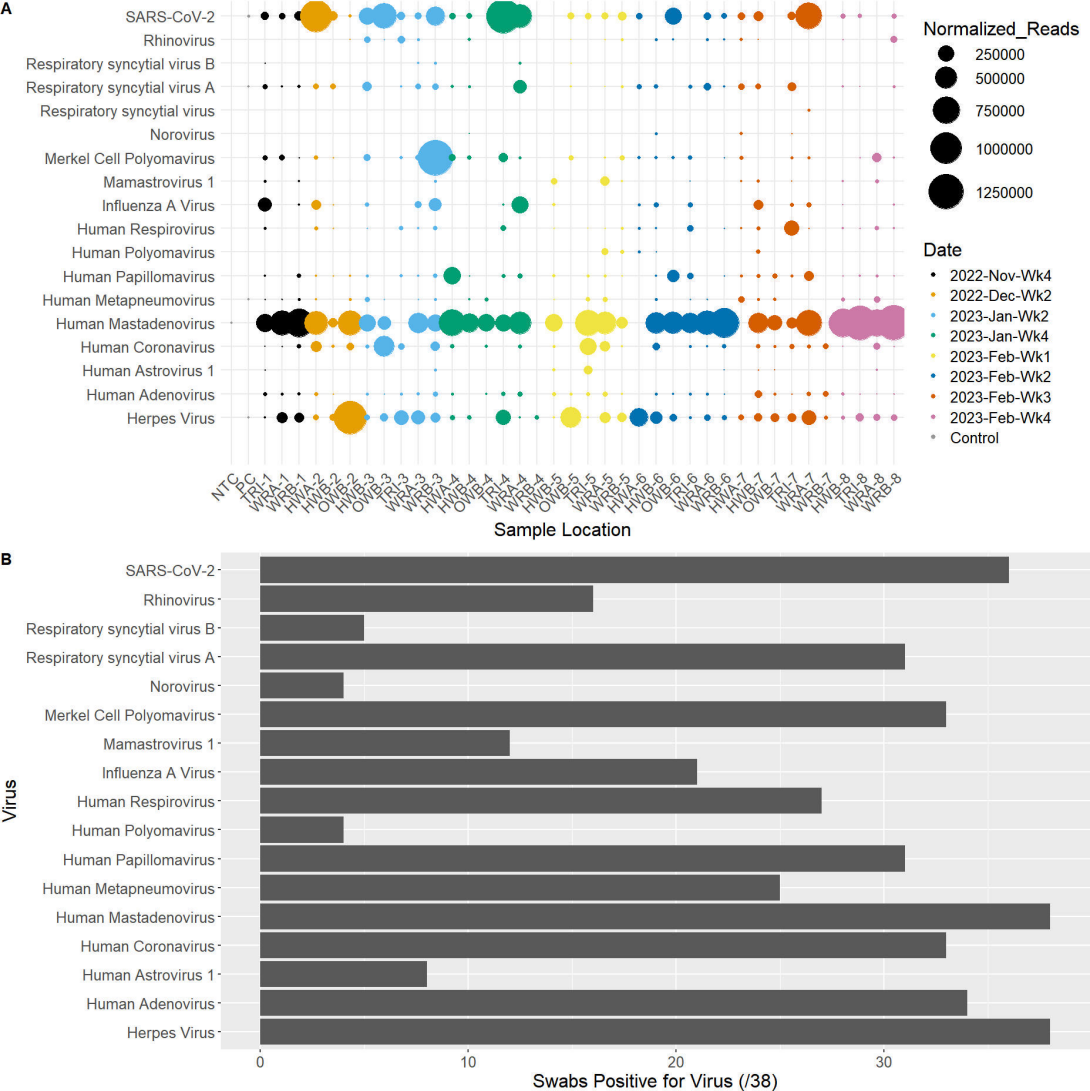
(**A**) Bubble chart showing the normalized read abundances of samples collected between the final week of November (week 4) 2022 and the final week of February (week 4) 2023 for all detected viruses in each sample. Samples are colored and ordered according to date, and the size of the bubble corresponds to abundance of normalized reads. HWA, hallway A; HWB, hallway B; NTC, no template control; OWB, outside waiting room B; PC, positive control; TRI, triage; WRA, waiting room A; WRB, waiting room B (a mixture of SARS-CoV-2, RSV, and influenza virus RNA). (**B**) Histogram showing the number of samples each virus was detected in (out of 38 swab samples). Samples were normalized using a scaling approach; the median number of viral target reads across all samples (not including controls) was determined, and a scaling factor for each sample was calculated compared to this median. The scaling factor was applied to all viral read counts within that sample.

Prior to computing the diversity of the viral populations in these samples, the read counts were normalized by scaling to the median read count across all samples (see Materials and Methods for further details); the scaled values were used to calculate all diversity statistics and were included in all diversity visualizations. Following the reduction of subtype variants, we found 18 viruses in our survey (Fig. 1A). Most viruses were detected in multiple samples, with a median of 27 of the 38 samples containing any given virus; none of the viruses were detected solely in a single sample (Fig. 1B). Several common respiratory pathogens were detected frequently, including SARS-CoV-2, influenza A, and RSV. Human mastadenovirus was found in all 38 samples and was consistently found in high abundance. Merkel cell polyomavirus was present in almost all samples (35/38) and both the positive control (defined viral community; described in Materials and Methods) and negative processing control (swab exposed to air only) at a low abundance, suggesting that the source for this virus may not be from the environment, but rather from airborne contamination or present in any of the sample processing workflow reagents.

**Fig 2:**
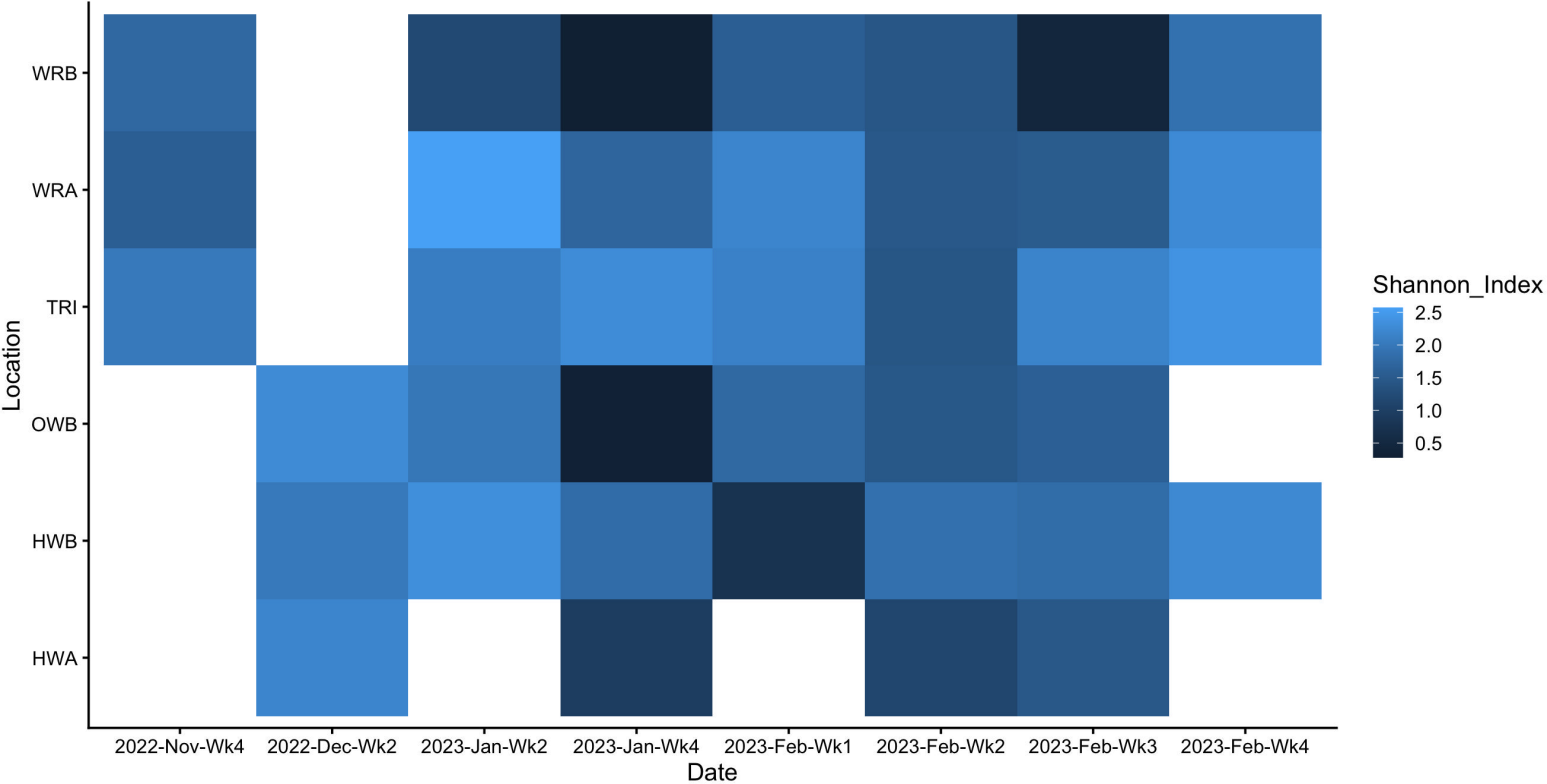
Shannon indices for 38 samples, collected from the final week of November 2022 to the final week of February 2023 in six locations within a regional hospital. White squares indicate that no sample was collected for that date-location combination. HWA, hallway A; HWB, hallway B; OWB, outside waiting room B; TRI, triage; WRA, waiting room A; WRB, waiting room B. Samples were normalized using a scaling approach; the median number of viral target reads across all samples (not including controls) was determined, and a scaling factor for each sample was calculated compared to this median. This scaling factor was applied to all viral read counts within that sample.

We assessed overall patterns of diversity of all viruses using the Shannon index (H), a measure of within-sample (alpha) diversity (Fig. 2). The negative control had a Shannon index of 0.0008, while the positive control had a Shannon index of 1.45. Samples taken on the same day generally had similar levels of diversity, and just over half of the samples had an index above 1. A two-way ANOVA was performed, and we found no significant difference in Shannon index by date or location (*F* (25) = 0.204, *P* > 0.5).

**Fig 3:**
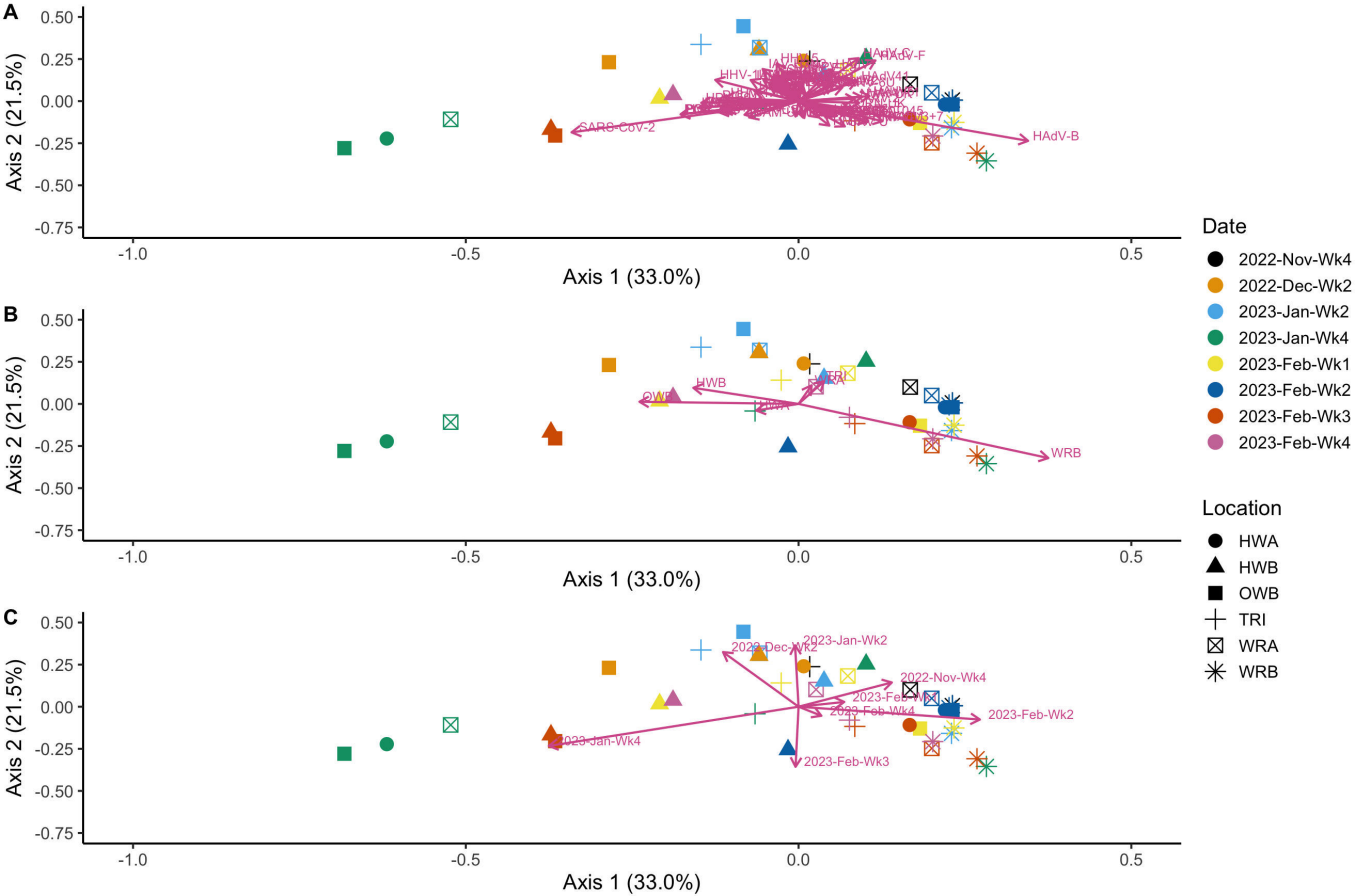
Principal coordinate analysis (PCoA) of the 38 swab samples. (**A**) PCoA for all viruses detected in the 38 samples. (**B**) PCoA for all samples by the location of sample collection. (**C**) PCoA for all samples by date of sample collection. HWA, hallway A; HWB, hallway B; OWB, outside waiting room B; TRI, triage; WRA, waiting room A; WRB, waiting room B. Samples were normalized using a scaling approach; the median number of viral target reads across all samples (not including controls) was determined, and a scaling factor for each sample was calculated compared to this median. This scaling factor was applied to all viral read counts within that sample.

Next, we assessed the extent to which pathogenic viral communities were conserved for samples collected from the same or similar locations, as well as for samples collected close in time, through principal coordinate analysis (Fig. 3). Overall, there is substantial clustering of the samples, indicative of shared community composition. Three viruses (human adenovirus [HAdV] B and F and SARS-CoV-2) showed strong influences on community composition, with vector arrows that extended away from the origin and perpendicular to one another (Fig. 3A). By location, WRB showed the strongest influence on the plot (Fig. 3B). Samples were similarly clustered by date, with week 1 of January 2023 being the most different (Fig. 3C). Using a PERMANOVA to test for the significance of date and location on diversity, we found the model (Y~Date + Location) to be significant (*F*_12,25_ = 2.50, *P* = 0.001) (Table 1). pairwiseAdonis was used to identify significant pairwise differences, which identified 4 out of 15 significant pairwise comparisons for location (Table 2).

**TABLE 1:** PERMANOVA results for beta-diversity^*a*^

	df	*F*-statistic	*P* value
Model	12	2.495	0.001
Residual	25		
Total	37		

^*a*^ The data used to compute the PERMANOVA were the viral target reads normalized to the median number of viral target reads across all samples.

**TABLE 2:** Pairwise comparisons following PERMANOVA^*a*^

Comparison by location	df	*F*-statistic	*P*-adj
WRB vs OWB	1	0.3086	0.015
WRB vs HWA	1	0.2681	0.045
WRB vs HWB	1	0.2758	0.015
WRB vs TRI	1	4.1931	0.045

^*a*^ Only significant comparisons are described in this table. HWA, hallway A; HWB, hallway B; OWB, outside waiting room B; TRI, triage; WRB, waiting room B.

### Variant analysis of SARS-CoV-2

**Fig 4:**
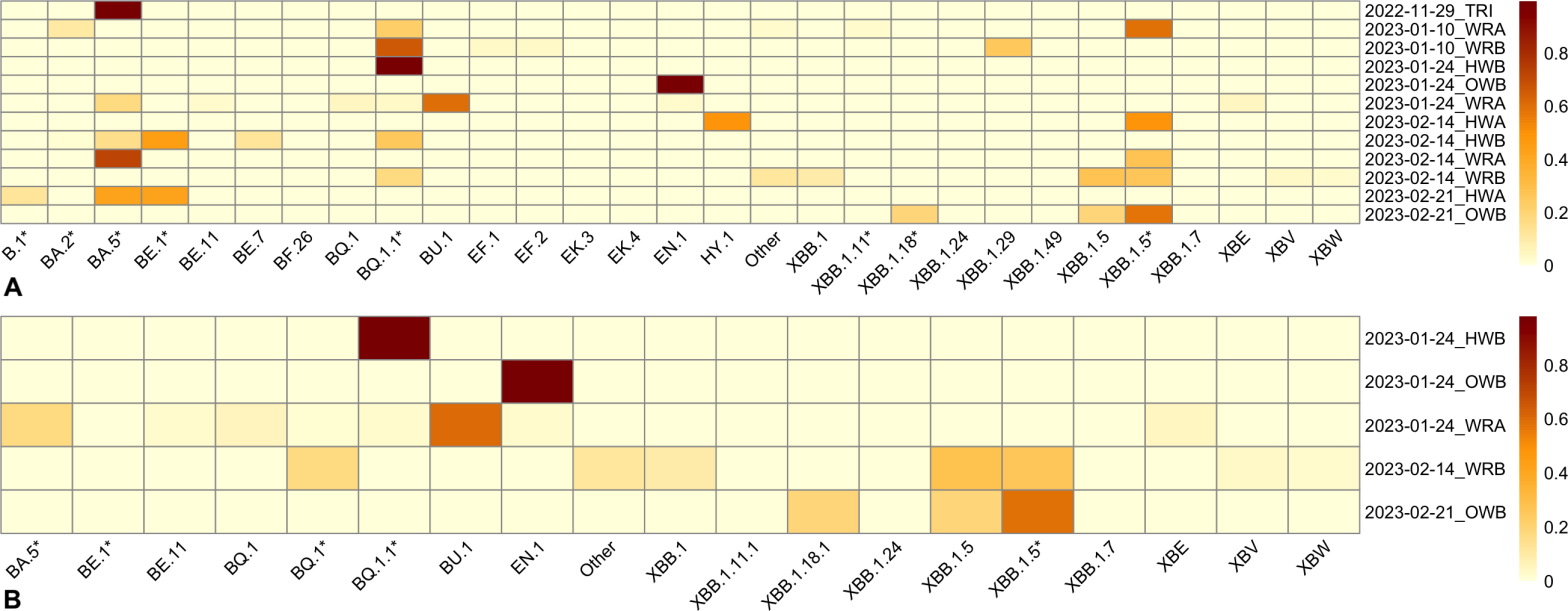
Heatmaps depicting SARS-CoV-2 variants’ relative abundance across samples, constrained to samples with percent genome coverage ≥60% (**A**) and ≥80% (**B**). SARS-CoV-2 variants are across the *x*-axis, with samples along the *y*-axis named as year-month-day_location. The heatmap shows lineages ordered alphabetically (*x*-axis) and samples are ordered chronologically (*y*-axis). Sublineages were collapsed based on shared numeric lineage nomenclature (denoted as *) when sublineages were in low abundance. All sublineages fall under the Omicron lineage, except for B.1* in panel **A**. HWA, hallway A; HWB, hallway B; NTC, no template control; OWB, outside waiting room B; PC, positive control; TRI, triage; WRA, waiting room A; WRB, waiting room B.

Following the extraction and analysis of SARS-CoV-2-only reads, we conducted a variant analysis. Samples had a wide range of read numbers assigned to SARS-CoV-2 (range: 6–29,777,128), which was also reflected in the ranges of percent SARS-CoV-2 genome covered (range: 1.97–99.89%) and mean coverage (range: 0.0258*x*−1.28e+05*x*) (Table S2). Samples with genome coverage values equal to and above 60% (*n* = 12) and 80% (*n* = 5) were included in these analyses (Fig. 4). We assessed the relative abundance of variants from each included sample along the facets of location and swab date (Fig. 4). For all samples using the ≥80% genome coverage cutoff, Omicron variants predominated throughout the sampling period (Fig. 4B). In this subset, there is a high abundance of BQ.1.1* and its sublineage EN.1 in the January samples, with a shift away from this lineage into XBB in February, with XBB.1.5 and its sublineages dominating by the second and third weeks of that month, and the appearance of XBB.1.18.1 in the sample collected in the third week of February. Other lineages, such as BA.5* and BU.1, also appear in moderate abundances at the end of January but are not prominent in the February samples. There was no apparent trend across locations, although the very small sample size (*n* = 5) hindered comparisons. Using the ≥60% coverage cutoff, all lineages belonged to the Omicron variant except for the ancestral B.1*, which is the ancestor to these SARS-CoV-2 lineages (Fig. 4A). BA.5 was most abundant in the sample from the final week of November 2022, whereas the five January 2023 samples had only its sublineage BQ.1.1 and no appreciable amount of BA.5. However, the results for both B.1* and BA.5 should be interpreted with caution, as reads belonging to sublineages could be misclassified by Freyja to higher level lineages rather than the appropriate sublineage. As observed in the ≥80% coverage subset, there is a shift into EN.1 at the end of January, which then shifts into XBB.1.5 and its sublineages. The analysis using the 80% cutoff is likely more accurate and reflective of the true changes in variants over the study period; reducing the threshold to 60% may have resulted in the inclusion of samples with less accurate strain detection, which may explain some of the differences observed in the overall variant trends across the sampling period.

### Read abundance as a measure of disease burden

In order to determine whether targeted metatranscriptomics provides an informative correlation with infection burden in the hospital setting, we investigated correspondence between targeted metatranscriptomics and markers of clinical disease burden for SARS-CoV-2, influenza, and RSV. Two relevant markers of disease burden were available: (i) hospital admissions associated with each virus provided a direct estimate of the number of patients admitted with severe infections in the facility; and (ii) qPCR analysis of floor samples with species-specific assays provided an independent estimate of viral RNA abundance and has been shown to correlate with clinical burden for SARS-CoV-2 (3, 34).

**Fig 5:**
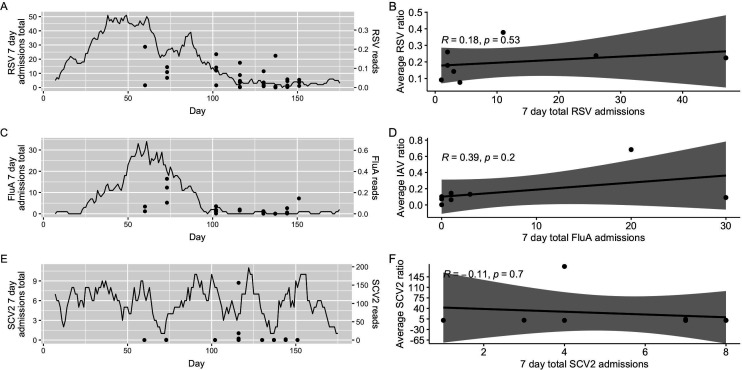
Comparison of the proportion of targeted metatranscriptomic read ratios relative to mastadenovirus with 7-day total hospital admissions for RSV (**A and B**), influenza A (FluA) (**C and D**), and SARS-CoV-2 (SCV2) (**E and F**). Data are plotted by date (**A, C, and E**) with the solid line depicting hospital admissions and data points (circles) showing read counts, and as a correlation between reads and admissions (**B, D, and F**). The proportion in these analyses was determined by assessing the relationship between the unscaled, original targeted metagenomic read counts for each virus to the mastadenovirus.

In these analyses, the read counts are represented as the proportion of reads belonging to the target virus relative to the number of reads classified as mastadenovirus for each sample. For SARS-CoV-2, influenza A, and RSV, we investigated the relationship between normalized sequencing read counts and total associated hospital admissions in 7 days leading up to the swab date (Fig. 5). We note that our analysis is limited to influenza A and omits influenza B, as only 2 out of 38 samples had reads (<500) for influenza B, and only one sample was qPCR-positive for this virus. Our sampling started during the second half of the fall peak of infections for RSV and influenza A and ended during a period of low hospital admissions (Fig. 5, panels A and C). For SARS-CoV-2, case counts were low throughout the sampling period (Fig. 5E). We did not find any significant correlations between read counts and cases for RSV (*R* = 0.18, *P* = 0.53), influenza A (*R* = 0.39, *P* = 0.2), or SARS-CoV-2 (*R* = −0.11, *P* = 0.7) when considering the ratio of raw reads for each of the three viruses relative to either the raw reads for mastadenovirus or the combined total of herpes virus reads (Fig. 5; Fig. S1).

**Fig 6:**
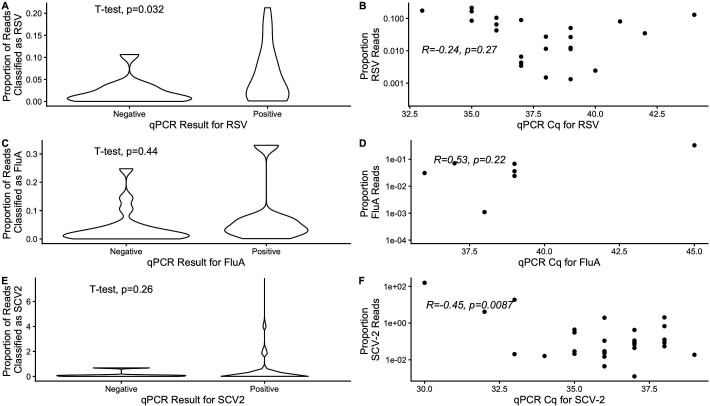
Relationship between the proportion of unnormalized targeted metagenomic read counts for each virus relative to mastadenovirus and virus-specific qPCR. Panels **A, C, and E** show read ratios of the target virus relative to mastadenovirus for samples that are qPCR-negative or qPCR-positive for RSV, influenza A (FluA), or SARS-CoV-2, respectively. Panels **B, D, and F** illustrate correlations between read count and qPCR Cq value for the positive samples. Reads in these analyses were normalized by assessing the relationship between the unscaled, original targeted metagenomic read counts for each virus to the mastadenovirus.

Similarly, we assessed whether targeted metatranscriptomics and qPCR give similar results when applied to the same floor swabs (Fig. 6). For all three viruses of interest, we compared the ratio of raw reads for each virus relative to the raw reads for mastadenovirus and found a statistically significant difference in metatranscriptomic read numbers between swabs that tested positive or negative by qPCR for RSV (*t*-test: *P* = 0.032) but not for SARS-CoV-2 or influenza A (Fig. 6A, C, and E). For swabs that tested positive by qPCR, we found the expected negative correlation between read number and Cq for SARS-CoV-2 (*R* = −0.45, *P* = 0.0087). Read number and Cq showed a weak negative correlation for RSV (*R* = −0.24, *P* = 0.27). For influenza A, read numbers appeared to show a non-significant positive correlation with Cq (*R* = 0.53, *P* = 0.22); however, upon removal of an apparent outlier at Cq = 45, there was no significant correlation between read numbers and Cq (*R* = −0.03, *P* = 0.96), although sample size was small (*n* = 7 swabs testing positive for influenza A by qPCR). We repeated this analysis using total reads belonging to herpes virus, rather than mastadenovirus, and found similar trends, except that there was no significant difference in the number of sequenced reads for RSV between qPCR-positive and qPCR-negative samples (Fig, S2). A similar analysis to compare the proportion of qPCR-positive samples to admissions showed no significant correlations for any of the three viruses (Fig. S3).

## DISCUSSION

In this study, we demonstrate that targeted metatranscriptomic sequencing can be used to characterize viral communities from hospital floors (Fig. 1). Despite the low nucleic acid yield typical of floor swab samples, we achieved adequate sequencing depth from all swabs assayed in this study, with a minimum of 2.2 million reads and a maximum of 30 million reads across all samples (Table S1). We found a variety of common (and therefore expected) viruses from hospital floors, including influenza, RSV, and SARS-CoV-2. Viral communities varied in the diversity of viruses present in the samples (Fig. 2). Date of collection and sampling location showed statistically significant differences in viral diversity, indicating that this method does capture meaningful differences across time and space. Similarly, we were able to identify the presence and proportion of different SARS-CoV-2 variants using these data, with a decrease in lineages such as BQ.1 and BQ.1.1 and increases in XBB.1 and XBB.1.5 lineages between November 2022 and February 2023 corresponding to known expansion times of those variants, particularly in Ontario and broadly through Canada (Fig. 4) (35, 36). Similarly, we have previously reported that floor samples can be used for SARS-CoV-2 variant characterization using targeted PCR amplification (37). However, the presence of some lineages, such as EN.1, BU.1, and XBB.1.18, in higher abundances during the sample period does not align with the same abundance data available for population spread at the time, suggesting potential misclassification rather than true presence.

To assess whether this method could be used as a correlate for disease burden, we compared the metatranscriptomic read counts with case burdens in the hospital and proportion of positive swabs for three viruses of interest. The results showed no significant correlation for each virus’s metatranscriptomic signal compared to the number of hospital admissions for disease related to any of influenza A, SARS-CoV-2, or RSV (Fig. 5B, D, and F). We found no correlation for SARS-CoV-2; however, we note that cases were fairly consistent and low in number over the study’s timeframe, making it difficult to compare to the viral sequencing signal with a smaller sample number (*n* = 36). The results for influenza A showed no correlation upon removal of an outlier data point, but the small sample size (*n* = 5) lacks the power to make definitive conclusions. Additional swabs would enhance the analysis presented here, including more replicates at each time point and increased frequency of collection, by potentially increasing positive swabs for all target viruses that could be included in this analysis.

We have previously shown that SARS-CoV-2 qPCR cycle threshold can be a useful predictor of COVID-19 burden, even when all swabs in a given facility test positive (making a presence/absence analysis impossible) (14). A previous study found that targeted amplicon sequencing depth correlated with qPCR signal for several targets in agricultural wastewater (38). We find modest correspondence between qPCR results and metatranscriptomic sequencing read counts. Swabs that were qPCR-positive for RSV showed higher RSV counts than qPCR-negative swabs (Fig. 6A). Moreover, we found the expected negative correlation between qPCR signal and read count for RSV and SARS-CoV-2 (Fig. 6B and F), although the correlation was weak and non-significant for RSV. Influenza A did not show significant correlation between read count and qPCR Cq in either direction, likely influenced by the small number of qPCR-positive swabs (*n*=7).

While the ability of meta-‘omic technologies to detect pathogens has been adequately demonstrated, our results gave a mixed verdict on whether sequencing data can accurately reflect the burden of disease, with lower power of prediction when case numbers were steady in the hospital. In wastewater, targeted metatranscriptomic sequencing has correlated with community-level disease burden for several viruses, including influenza, SARS-CoV-2, and monkeypox virus (39). This relationship is less clear in our study, where we found positive, yet non-significant, correlations between viral reads and hospital admissions. However, we did find some correspondence between qPCR signal and read depth for RSV and SARS-CoV-2, suggesting that increased viral burden would be reflected by both an increased number of sequenced reads and fewer qPCR cycles necessary to reach an appropriate threshold.

A major benefit of targeted metatranscriptomics is the elimination of non-target genetic material that would be sequenced using shotgun metagenomics, as viral genetic material may make up only a small fraction of the genetic material collected from each sample (27). In our study, the floor swabs would have captured the genetic material from patients, other organisms in the environment, as well as commensal bacteria. Several studies have demonstrated increased sensitivity for targeted approaches in clinical samples (28) and in wastewater (27, 29). We thus expect a substantial improvement in sensitivity for targeted sequencing compared with shotgun sequencing, which will be explored in future trials now that the detection capacity of targeted metatranscriptomics has been established. However, since many targeted sequencing panels focus on human pathogens, non-pathogen viral targets are likely to be missed and not accounted for in diversity measures. A combination of both targeted and non-targeted viral sequencing may better capture overall viral diversity in the built environment (27, 40).

Limitations to this method of viral surveillance include sample collection inconsistency, sample location selection, sample size, RNA extraction efficiency, and target mismatches to emerging variants of interest. First, while sample collection followed our established protocol, there may have been some variability that impacted the volume of material collected per swab. Similarly, the choice of sample location may also impact which viruses are detected; that is, whether the area is high- or low-traffic, or whether it is a space where someone who is shedding the virus is likely to contribute to the floor sample. Second, a small sample size (*n* = 38) was used to assess multiple time points and locations; a larger number of samples would strengthen this analysis and might help identify broader trends related to the change in viral load over time, as well as identify differences between locations. Next, a targeted metatranscriptomics approach is sensitive to the method of nucleic acid extraction used to obtain the viral nucleic acids. The yield of genetic material can vary greatly depending on the method used, which can impact the starting amount of material used for the enrichment and targeted sequencing of the samples (41). There is evidence that with targeted sequencing panels, there may be uneven coverage of targets, particularly regions with variations in GC content (42), and the processing of samples for sequencing can also impact which sequences are ultimately captured and sequenced (43). As such, results from these panels may not fully capture the total diversity of viruses for which the panel was designed to detect. Finally, while the Qiagen xHyb Adventitious Agent Panel comprises 132 potential viral targets (44), viruses or variants absent from the panel may be missed, and there is the potential that emerging variants will not be detected, or be detected at lower efficiencies due to out-of-date probes. Some researchers have shown that hybrid capture methods may result in collecting ambiguous reads that can be assigned to incorrect viruses (45). Within the data analysis, viruses may be identified at the family, virus, or variant levels depending on the sequenced region and composition of the reference database used for identification. Emerging variants may show a loss of sensitivity for identification and prevalence calculations depending on the probe library used for targeting.

While previous studies have shown promise for combining targeted metagenomic sequencing with surveillance and infection control, our results may suggest that there are limitations to this application (46). However, with more robust testing and assessment for the applications where targeted metagenomic sequencing is most effective, this approach could be used to improve infection control and surveillance. This method can additionally be used for identification of resistant and/or high-consequence pathogens, ascertainment of infection risk for nosocomial transmission, and determining overall facility burden. Similarly, these results show some promise in tracking the evolution of outbreaks, particularly in high-risk areas during a pandemic, but more work will be necessary to ascertain the full utility and applicability of such findings. Moreover, the ability to continuously expand the suite of detection targets, combined with the efficiency of the workflow and analysis pipelines, suggests this approach could be readily adapted for emerging pathogens and future outbreaks and epidemics.

Here, we evaluated the effectiveness of a targeted meta-’omics approach for reflecting the disease burden of a suite of viruses simultaneously, including influenza A virus, RSV, and SARS-CoV-2 in the built environment of an institutional healthcare setting. We identified some correlation between the number of reads and qPCR positivity, but we were largely unable to correlate reads with disease burden. Interestingly, we were able to track changes in SARS-CoV-2 variant relative abundances over the course of the study using targeted sequencing reads, suggesting that such analyses could be applied to other viruses, particularly in the event of outbreaks. Continued work to evaluate the accuracy of disease burden prediction from metatranscriptomic-based environmental surveillance is required, including tracking correlations through high and low instances of hospitalization for each virus of interest.

## MATERIALS AND METHODS

### Study design

As part of a large, city-wide evaluation of built environments for viral infection surveillance, we collected floor samples prospectively over a 4-month period from a large tertiary care center emergency department in Ottawa, Canada to evaluate the relationship between built environment viral detection and overall viral infection burden (47). During the study period, infection prevention and control measures in the emergency department included universal masking among patients, family, and healthcare workers; visitor restrictions and reduction of family caregivers to one adult accompanying the patient; physical distancing in waiting rooms to 1 m between seats. Daily admission counts for patients with RSV, influenza A, or SARS-CoV-2 during the study period were routinely collected and submitted to the Ontario Ministry of Health. The University of Ottawa deemed this project exempt from formal research ethics board review.

### Sample collection

Targeted sequencing was performed on a subset of weekly samples (*n* = 38) collected between November 2022 and February 2023 from common areas within the hospital emergency department, including the triage area of the emergency department (TRI), two waiting rooms (waiting room A [WRA] and waiting room B [WRB]), the foyer outside WRB (OWB), and the hallways outside of two exam rooms (hallway A [HWA] and hallway B [HWB]) (47). These samples were collected for a previous study exploring city-wide SARS-CoV-2 prevalence in the built environment (47). At each location, a 2 in. × 2 in. area of floor was swabbed using the P-208 Environmental Surface Collection Prototype kit from DNA Genotek. Swabs were immersed in 1 mL nucleic acid stabilization solution and collection vials were stored for approximately 1–2 weeks at room temperature during transport and prior to laboratory processing. Controls included a negative processing control (swab of air in laboratory) and a positive RNA control consisting of 200 copies/µL each of influenza A, influenza B, RSV, and SARS-CoV-2 RNA standards. Influenza A, influenza B, and SARS-CoV-2 synthetic RNA controls were obtained from Twist Bioscience (catalog numbers 103001, 103003, and 103907, respectively). The RSV control was quantified genomic RNA from RSV strain A2 (ATCC VR-1540 DQ), obtained from Cedarlane Laboratories.

### Nucleic acid extraction and qPCR

Total nucleic acids were extracted from 300 µL of stabilization solution using the MagMAX Viral/Pathogen II (MVP II) Nucleic Acid Isolation Kit (Thermo Fisher), as previously described (8). A 5 µL of the 50 µL total eluate was analyzed by qPCR for the presence of iinfluenza A, influenza B, SARS-CoV-2, and RSV using a custom multiplex Taqman assay. The assay combines primers and probe targeting RSV (both type A and type B) (48) with the influenza A, influenza B, and SARS-CoV-2-targeting components of the CDC Influenza SARS-CoV-2 (Flu SC2) Multiplex Assay (49). Table S3 lists the sequences and concentrations of the oligonucleotide primers and probes in the multiplex assay.

Multiplex qPCR detection was carried out in 20 µL reactions consisting of 5 µL of 4× TaqPath 1-Step Multiplex Master Mix (No ROX) (Thermo Fisher), 1 µL of 20× multiplex primer/probe mix, 9 µL water, and 5 µL of template. Real-time PCR was performed on a Bio-Rad CFX Opus 96 system with the following cycling conditions: 25°C for 2 min, 50°C for 15 min, 95°C for 2 min, followed by 45 amplification cycles (95°C for 15 s and 55°C for 30 s). Excitation and detection of fluorophores used channels 1–4 with the following selected fluorophores: FAM, VIC (for Yakima Yellow), Texas Red, and CY5. Cq values were determined with Bio-Rad CFX Maestro software with baseline subtraction based on cycles 5–15 and a signal threshold of 75 for each target. The multiplex assay was validated against the four above RNA standards. Swab positivity was determined with a Cq cutoff of 45 cycles, and no template controls were consistently negative for all four targets at this cutoff.

### Targeted sequencing

Following reverse transcription, sequencing libraries were prepared using the Qiagen FX DNA library kit according to the manufacturer’s protocol. Libraries were pooled prior to hybridization, with up to 1,500 ng of DNA per pool; sample inputs varied depending on library yield, as swab samples from surfaces can provide low biomass. Pooled libraries were subjected to hybridization capture using the Qiagen xHyb Adventitious Agent panel and PCR amplified for 7–10 cycles. This bait-capture panel includes probes for 100 viral targets (44), including subtypes for some viruses (notably influenza). Sequencing was then carried out on an Illumina NextSeq550. All reads were analyzed using Kraken2 v2.1.3 using the standard RefSeq database (k2_standard_20230605 database) (50), and Kraken Tools v1.2 (51) was used to retain reads identified as viral, microbial, and fungal in origin (ENA study accession PRJEB106823).

### Bioinformatic analysis

Following sequencing, the fastq files were analyzed using FastQC v0.12.1 (https://www.bioinformatics.babraham.ac.uk/projects/fastqc/) for initial quality control. The samples were then trimmed using Trimmomatic v0.39 (http://www.usadellab.org/cms/?page=trimmomatic) using the following parameters: LEADING:3 TRAILING:3 MINLEN:36. Data files were analyzed using the Qiagen-provided QIAseq xHYB Microbial analysis module (https://geneglobe.qiagen.com/us/analyze). The trimmed fastq files were uploaded and processed under default parameters using the options “Next-Generation Sequencing” for analysis type, “Microbial” for analyte, and “QIAseq xHyb” for the panel. Briefly, samples were further trimmed within the module to remove low-quality reads, and the taxonomic profile was determined for each sample, including the abundances of reads associated with each virus in the panel. Note that the module included a step to remove duplicate mapped reads which may have arisen from amplifying the genetic material in the library preparation steps and may not be reflective of the true abundance of those reads in the original sample. The abundances for each virus in each sample were recorded in a pivot table and used for further analyses.

### Data analysis

The sequenced reads were normalized prior to use in analyses to control for different data set sizes. Normalization was done by assessing the median number of reads mapping to the viral targets for all 38 samples, omitting the negative processing control and the positive control with known viral composition. All samples were scaled such that the sum of the newly normalized read counts was equal to this median value. This can be summarized by the following equation: normalized read count per target = (sample A read count for target virus) × (total read count for sample A/median read count for all samples); this is repeated on all targets in sample A and computed for each subsequent sample. Any samples which had a total read count below the read count of the negative control were to be excluded from use for further analyses; no samples fell below this threshold. For most analyses, read numbers from viral subtypes were collapsed into a single count for a virus; separated viral counts for all subtypes were used for diversity analyses (described below). All virus abundances were visualized over time and location, as well as correlated to known hospital admissions for three specific viruses (influenza A, RSV, and SARS-CoV-2) using ggplot2 (v.3.5.1) (52). Alpha- and beta-diversity were assessed using the R package vegan (2.6.6.1) (53). A PERMANOVA was calculated for beta-diversity using adonis in vegan, and pairwise comparisons were done using pairwiseAdonis (0.4.1) (54). To better compare the relationship of specific viral reads to hospital admissions for the three viruses, we used the ratio of reads for the focal virus to the number of reads for mastadenovirus for each sample, rather than the normalized number of reads per sample for the focal virus. Human mastadenovirus was chosen as it is present in fairly stable quantities across all samples (but not in the controls). Similarly, we also used the ratio of reads for the focal virus to the number of reads to the combined number of reads for all varieties of herpes virus, another virus that was present in stable quantities.

For the analysis of SARS-CoV-2 variants, Kraken2 (v.2.1.3) (50) and KrakenTools (v.1.2) (51) were used to extract SARS-CoV-2-specific reads. SARS-CoV-2 reads were aligned to the default SARS-CoV-2 genome (NC_045512_Hu-1) using minimap (v.2.28) (55) and sorted using samtools (v.1.21) (56). Freyja (v.1.5.3) (57) was then used following alignment to identify variants and their relative abundances within each sample. samtools coverage was also used to compute the percent genome covered and mean coverage. Samples with percent genome covered values below 60% were discarded from further analysis. For readability within figures, sublineages were in some cases condensed; for example, sublineages XBB.1.5.19 and XBB.1.5.60 were denoted as XBB.1.5*, rather than as separate sublineages. Note: lineages and sublineages were collapsed solely on the basis of shared naming convention of the lineage number, and not collapsed by sublineages that fall beneath the umbrella of a specific lineage. The results were then visualized using pheatmap (v.1.0.13) in R (v.4.4.2) using both a ≥60% and an ≥80% threshold (58)).

## SUPPLEMENTAL MATERIAL

Supplemental material msphere.00086-26-s0001.docx Figures S1-S3; Tables S1-S3.

ASM does not own the copyrights to Supplemental Material that may be linked to, or accessed through, an article. The authors have granted ASM a non-exclusive, world-wide license to publish the Supplemental Material files. Please contact the corresponding author directly for reuse.
