# Supplementary material for: Targeted metatranscriptomic detection of viruses from floors for simultaneous evaluation of respiratory disease burden and viral variant identification
Source: mSphere. 2026 Apr 20;11(5):e00086-26. doi: 10.1128/msphere.00086-26 (PMC13203961; doi:10.1128/msphere.00086-26)
Supplement: Supplemental material — Figures S1-S3; Tables S1-S3. [file msphere.00086-26-s0001.docx]

**Additional Figures**


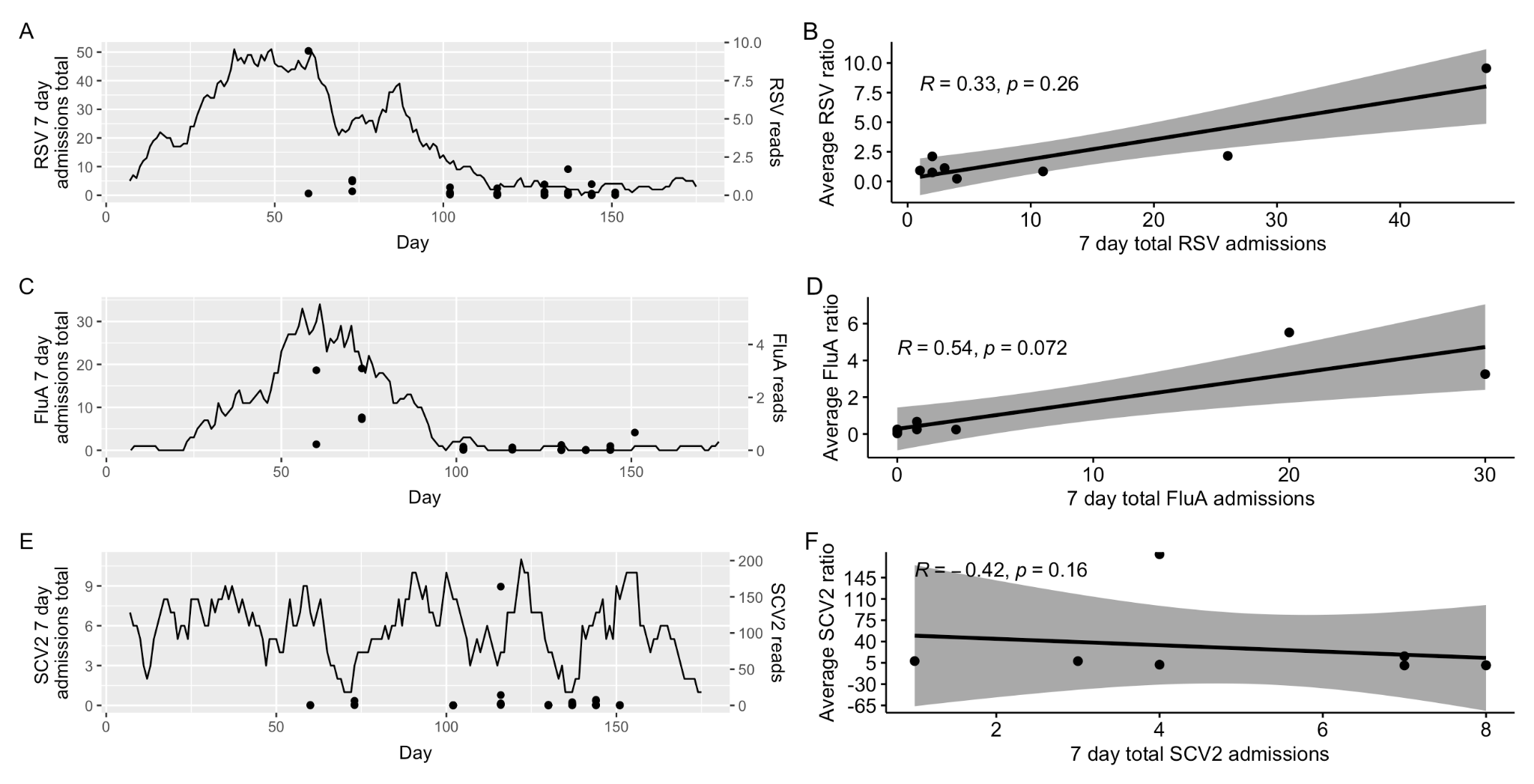


**Figure S1.** Comparison between the proportion of unnormalized targeted metatranscriptomic read ratios relative to combined herpes virus and 7-day total hospital admissions for RSV (A, B), Influenza A (FluA) (C, D), and SARS-CoV-2 (E, F). Data are plotted by date (A, C, E) with the solid line depicting hospital admissions and data points (circles) showing read counts, and as a correlation between reads and admissions (B, D, F).


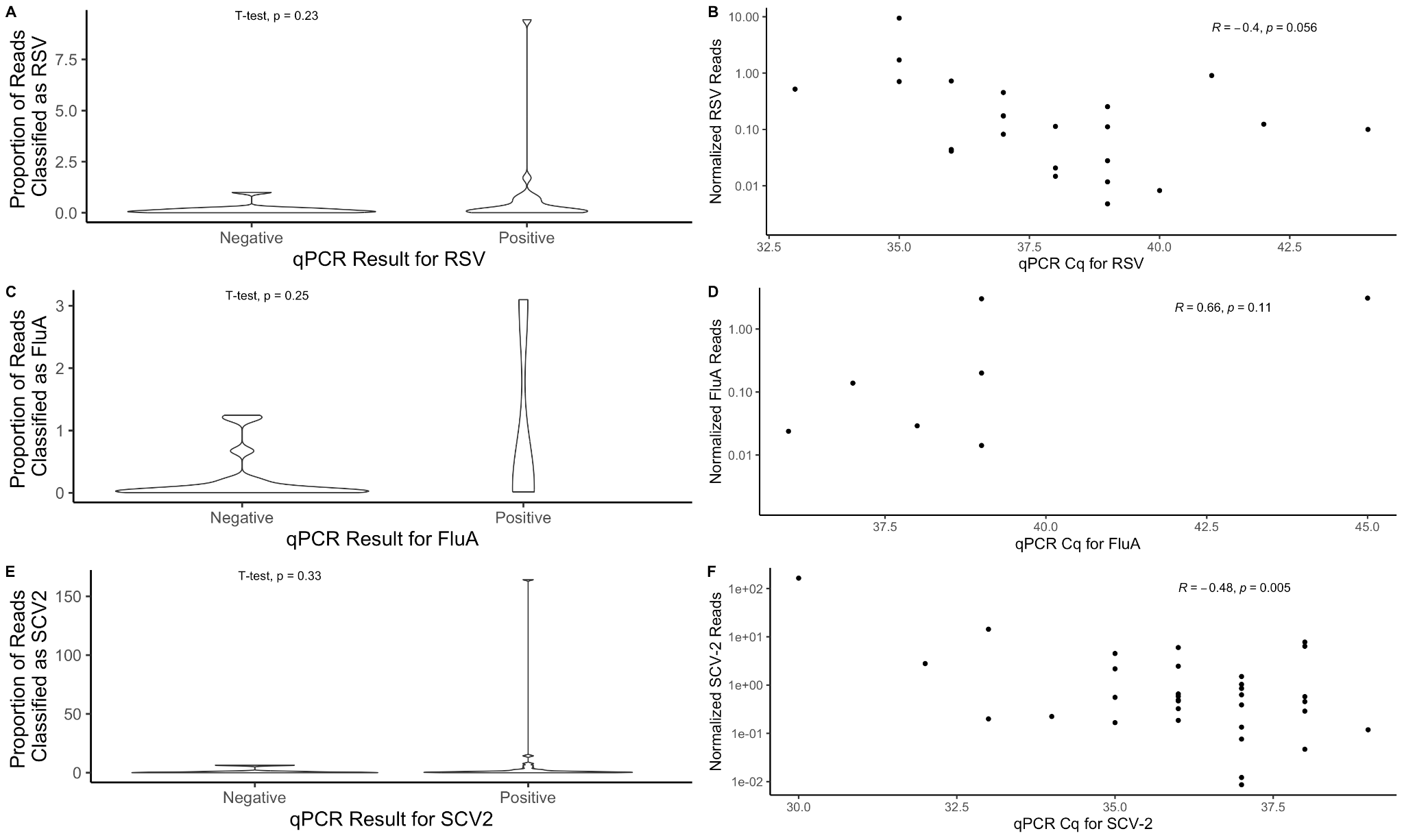


**Figure S2:** Relationship between the proportion of unnormalized targeted metagenomic read counts for each virus relative to the total amount of herpes virus per sample and virus-specific qPCR. Panels A, C, and E show normalized read counts for samples that are qPCR negative or positive for RSV, Influenza A (FluA), or SARS-CoV-2, respectively. Panels B, D, and F illustrate correlations between read count and qPCR Cq value for the positive samples.


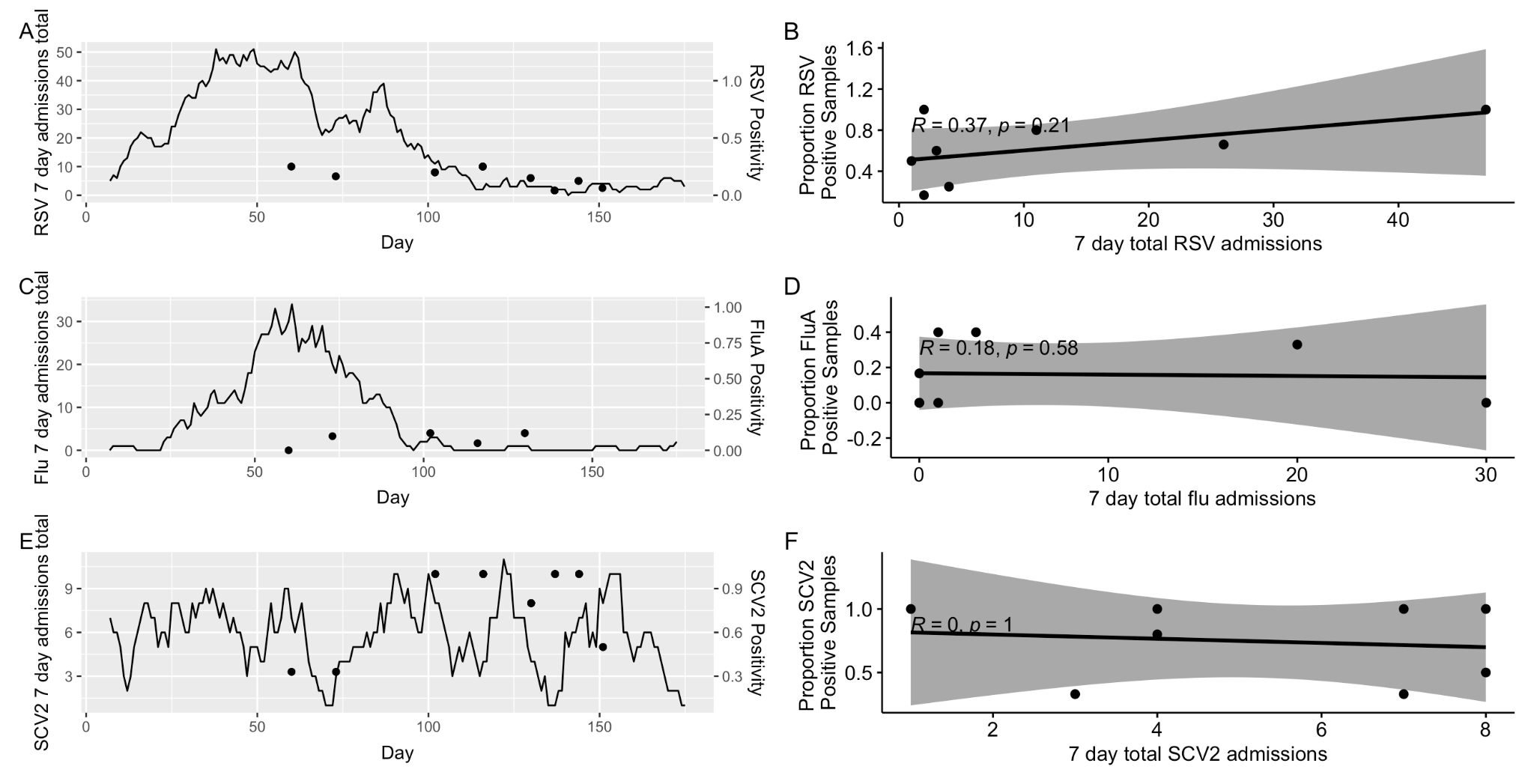


**Figure S3.** Comparison between the proportion of positive samples, as determined by qPCR, and 7-day total hospital admissions for RSV (A, B), Influenza A (FluA) (C, D), and SARS-CoV-2 (E, F). Data are plotted by date (A, C, E) with the solid line depicting hospital admissions and data points (circles) showing the proportion of positive samples, and as a correlation between the proportion of positive samples and admissions (B, D, F).

**Table S1:** Raw number of reads from a targeted meta-’omics approach for 38 samples (excluding controls). Samples were assessed for the total number of viral reads, with viruses matching to the viral panel of the xHyb Adventitious Agent Panel. WRA = Waiting Room A, WRB = Waiting Room B, OWB = Outside Waiting Room B, HWA = Hallway A, HWB = Hallway B, TRI = Triage.

| Sample | Total Number of Reads (raw) | Total Number Viral Mapped Reads |
| --- | --- | --- |
| 2022-DecWk2_HWA | 3,068,112 | 902,566 |
| 2023-JanWk2_HWA | 2,980,184 | 465,870 |
| 2023-JanWk4_HWA | 3,648,474 | 573,516 |
| 2023-FebWk1_HWA | 10,035,418 | 4,857,494 |
| 2023-FebWk2_HWA | 4,618,296 | 595,178 |
| 2023-FebWk3_HWA | 4,882,338 | 1,027,290 |
| 2023-FebWk4_HWA | 5,574,322 | 1,493,508 |
| 2022-DecWk2_HWB | 5,024,876 | 1,714,294 |
| 2023-JanWk4_HWB | 8,058,616 | 4,438,720 |
| 2023-FebWk2_HWB | 2,801,104 | 948,134 |
| 2023-FebWk3_HWB | 4,812,420 | 1,678,384 |
| 2022-DecWk2_OWB | 2,299,242 | 832,056 |
| 2023-JanWk2_OWB | 3,668,258 | 538,682 |
| 2023-JanWk4_OWB | 30,550,722 | 24,509,828 |
| 2023-FebWk1_OWB | 2,410,312 | 506,880 |
| 2023-FebWk2_OWB | 5,120,670 | 2,256,760 |
| 2023-FebWk3_OWB | 4,860,840 | 1,316,500 |
| 2022-NovWk4_TRI | 9,760,400 | 4,690,328 |
| 2023-JanWk2_TRI | 5,733,018 | 761,222 |
| 2023-JanWk4_TRI | 4,340,890 | 385,904 |
| 2023-FebWk1_TRI | 3,404,714 | 455,134 |
| 2023-FebWk2_TRI | 10,607,000 | 6,389,984 |
| 2023-FebWK3_TRI | 5,957,812 | 737,474 |
| 2023-FebWk4_TRI | 3,403,286 | 497,870 |
| 2022-NovWk4_WRA | 9,579,450 | 3,140,420 |
| 2023-JanWk2_WRA | 6,479,528 | 2,329,138 |
| 2023-JanWk3_WRA | 6,814,664 | 2,595,362 |
| 2023-FebWk1_WRA | 4,541,930 | 1,289,264 |
| 2023-FebWk2_WRA | 4,054,486 | 331,180 |
| 2023-FebWk3_WRA | 3,321,850 | 486,578 |
| 2023-FebWk4_WRA | 3,809,240 | 592,984 |
| 2022-NovWk4_WRB | 9,484,550 | 4,628,042 |
| 2023-JanWk2_WRB | 3,458,470 | 507,644 |
| 2023-JanWK3_WRB | 8,926,076 | 3,493,672 |
| 2023-FebWk1_WRB | 5,473,984 | 1,923,138 |
| 2023-FebWk2_WRB | 13,971,306 | 8,786,832 |
| 2023-FebWk3_WRB | 21,910,990 | 18,102,206 |
| 2023-FebWk4_WRB | 5,120,698 | 1,627,976 |

**Table S2.** Reads assigned as SARS-CoV-2 for each sample, including percent of genome covered and mean coverage.

| Sample | Reads Classified as SARS-CoV-2 | Percent Covered | Mean Coverage |
| --- | --- | --- | --- |
| 2022-11-Wk5_WRB | 5187 | 8.67% | 21.2x |
| 2022-12-Wk2_OWB | 272467 | 16.48% | 1.18e+03x |
| 2023-01-Wk2_OWB | 7429 | 2.17% | 30.3x |
| 2023-01-Wk4_HWB | 4450150 | 99.67% | 1.95e+04x |
| 2023-02-Wk1_HWA | 46051 | 19.34% | 197x |
| 2023-02-Wk2_HWA | 164916 | 71.44% | 700x |
| 2023-02-Wk3_TRI | 30351 | 19.75% | 132x |
| 2023-02-Wk4_WRA | 44415 | 12.34% | 199x |
| 2022-11-Wk5_WRA | 162 | 0.53% | 0.704x |
| 2023-01-Wk2_WRB | 11812 | 69.03% | 47.6x |
| 2023-01-Wk4_WRB | 18877 | 31.16% | 75.1x |
| 2023-01-Wk4_OWB | 29777128 | 99.89% | 1.28e+05x |
| 2023-02-Wk1_OWB | 14045 | 3.43% | 55.9x |
| 2023-02-Wk2_HWB | 30559 | 65.80% | 131x |
| 2023-02-Wk3_HWA | 584549 | 68.70% | 2.55e+03x |
| 2023-02-Wk4_TRI | 32835 | 12.30% | 136x |
| 2022-11-Wk5_TRI | 57799 | 65.45% | 250x |
| 2023-01-Wk2_WRA | 153681 | 79.40% | 623x |
| 2023-01-Wk4_WRA | 1819803 | 99.64% | 7.81e+03x |
| 2023-02-Wk1_WRB | 44641 | 34.05% | 181x |
| 2023-02-Wk2_WRB | 151971 | 92.33% | 651x |
| 2023-02-Wk2_OWB | 55545 | 18.81% | 226x |
| 2023-02-Wk3_HWB | 129812 | 16.22% | 558x |
| 2023-02-Wk4_HWA | 88480 | 19.77% | 360x |
| 2022-12-Wk2_HWA | 61363 | 8.32% | 255x |
| 2023-01-Wk2_TRI | 27362 | 32.92% | 113x |
| 2023-01-Wk4_TRI | 98247 | 36.27% | 422x |
| 2023-02-Wk1_WRA | 57954 | 16.74% | 245x |
| 2023-02-Wk2_WRA | 8899 | 61.30% | 38.1x |
| 2023-02-Wk3_WRB | 99070 | 57.25% | 397x |
| 2023-02-Wk3_OWB | 866561 | 88.30% | 3.71e+03x |
| 2022-12-Wk2_HWB | 89331 | 15.52% | 409x |
| 2023-01-Wk2_HWA | 3930 | 18.30% | 17x |
| 2023-01-Wk4_HWA | 6 | 1.97% | 0.0258x |
| 2023-02-Wk1_TRI | 20043 | 25.78% | 83.1x |
| 2023-02-Wk2_TRI | 206976 | 47.57% | 889x |
| 2023-02-Wk3_WRA | 23667 | 11.96% | 99x |
| 2023-02-Wk4_WRB | 29277 | 8.43% | 114x |

**Table S3.** Components of multiplex qPCR assay targeting Influenza A, Influenza B, SARS-CoV-2, and respiratory syncytial virus.

| **Name** | **Target gene or region** | **Oligonucleotide sequence (5' to 3')** | **Concentration (µM)^1^** | **Reference** |
| --- | --- | --- | --- | --- |
| ***Primers^2^*** |  |  |  |  |
| CDC_InfA For1 | Influenza A (matrix protein) | CAA GAC CAA TCY TGT CAC CTC TGA C | 3.33 | (Shu et al. 2021) |
| CDC_InfA For2 | Influenza A (matrix protein) | CAA GAC CAA TYC TGT CAC CTY TGA C | 3.33 | (Shu et al. 2021) |
| CDC_InfA Rev1 | Influenza A (matrix protein) | GCA TTY TGG ACA AAV CGT CTA CG | 5 | (Shu et al. 2021) |
| CDC_InfA Rev2 | Influenza A (matrix protein) | GCA TTT TGG ATA AAG CGT CTA CG | 1.67 | (Shu et al. 2021) |
| CDC_InfB For | Influenza B (nonstructural protein) | TCC TCA AYT CAC TCT TCG AGC G | 6.67 | (Shu et al. 2021) |
| CDC_InfB Rev | Influenza B (nonstructural protein) | CGG TGC TCT TGA CCA AAT TGG | 6.67 | (Shu et al. 2021) |
| CDC_SC2 For | SARS-CoV-2 (Nucleoprotein–3′ untranslated region) | CTG CAG ATT TGG ATG ATT TCT CC | 6.67 | (Shu et al. 2021) |
| CDC_SC2 Rev | SARS-CoV-2 (Nucleoprotein–3′ untranslated region) | CCT TGT GTG GTC TGC ATG AGT TTA G | 6.67 | (Shu et al. 2021) |
| RSV_Forward | Respiratory syncytial virus (matrix protein) | GGC AAA TAT GGA AAC ATA CGT GAA | 6.67 | (Fry et al. 2010) |
| RSV_Reverse | Respiratory syncytial virus (matrix protein) | TCT TTT TCT AGG ACA TTG TAY TGA ACA G | 6.67 | (Fry et al. 2010) |
| ***Probes^3^*** |  |  |  |  |
| CDC_InfA-P^4^ | Influenza A (matrix protein) | 56-FAM/TGC AGT CCT /ZEN/ CGC TCA CTG GGC ACG/3IABkFQ | 1.67 | (Shu et al. 2021) |
| CDC_InfB-P^5^ | Influenza B (nonstructural protein) | 5YakYel/CCA ATT CGA /ZEN/ GCA GCT GAA ACT GCG GTG/3IABkFQ | 1.67 | (Shu et al. 2021) |
| CDC_SC2-P^6^ | SARS-CoV-2 (Nucleoprotein–3′ untranslated region) | 5TexRd-XN/ATT GCA ACA /TAO/ ATC CAT GAG CAG TGC TGA CTC/3IAbRQSp | 1.67 | (Shu et al. 2021) |
| RSV_Cy5_P^7^ | Respiratory syncytial virus (matrix protein) | 5Cy5/CTG TGT ATG /TAO/ TGG AGC CTT CGT GAA GCT/3IAbRQSp | 1.67 | Modified from Fry et al. (2010) |

^1^ The concentration of each oligonucleotide in the 20X primer/probe mix for the multiplex assay.

^2^ Primers were obtained from Thermo Fisher using the custom DNA oligonucleotide synthesis service.

^3^ Probes were obtained from Integrated DNA Technologies (IDT) with the following catalogue numbers: 10011561 (Influenza A probe), 10011564 (Influenza B probe), 10011567 (SARS-CoV-2 probe). The RSV probe was synthesized as an IDT custom PrimeTime qPCR probe, based on sequences reported by Fry et al. (2010). For multiplex compatibility, the probe was modified to include a Cy5 reporter and appropriate quenchers.

^4^ The InfA probe was labeled with a 5’ 6-carboxyfluorescein reporter (56-FAM), an internal ZEN quencher, and a 3’ Iowa Black FQ quencher

^5^ The InfB probe was labeled with a 5’ Yakima Yellow reporter (5YakYel), an internal ZEN quencher, and a 3’ Iowa Black FQ quencher

^6^ The SC2 probe was labeled with a 5’ Yakima Yellow reporter (5TexRd-XN), an internal TAO quencher, and a 3’ Iowa Black RQ quencher

^7^ The RSV probe was labeled with a 5’ Cy5 reporter (5Cy5), an internal TAO quencher, and a 3’ Iowa Black RQ quencher
